# Combining botanical collections and ecological data to better describe plant community diversity

**DOI:** 10.1371/journal.pone.0244982

**Published:** 2021-01-07

**Authors:** Christina Alba, Richard Levy, Rebecca Hufft

**Affiliations:** Research & Conservation Department, Denver Botanic Gardens, Denver, Colorado, United States of America; Qinghai University, CHINA

## Abstract

In this age of rapid biodiversity loss, we must continue to refine our approaches to describing variation in life on Earth. Combining knowledge and research tools from multiple disciplines is one way to better describe complex natural systems. Understanding plant community diversity requires documenting both pattern and process. We must first know which species exist, and where (i.e., taxonomic and biogeographic patterns), before we can determine why they exist there (i.e., ecological and evolutionary processes). Floristic botanists often use collections-based approaches to elucidate biodiversity patterns, while plant ecologists use hypothesis-driven statistical approaches to describe underlying processes. Because of these different disciplinary histories and research goals, floristic botanists and plant ecologists often remain siloed in their work. Here, using a case study from an urban greenway in Colorado, USA, we illustrate that the collections-based, opportunistic sampling of floristic botanists is highly complementary to the transect- or plot-based sampling of plant ecologists. We found that floristic sampling captured a community species pool four times larger than that captured using ecological transects, with rarefaction and non-parametric species estimation indicating that it would be prohibitive to capture the “true” community species pool if constrained to sampling within transects. We further illustrate that the discrepancy in species pool size between approaches led to a different interpretation of the greenway’s ecological condition in some cases (e.g., transects missed uncommon cultivated species escaping from nearby gardens) but not others (e.g., plant species distributions among functional groups were similar between species pools). Finally, we show that while using transects to estimate plant relative abundances necessarily trades off with a fuller assessment of the species pool, it is an indispensable indicator of ecosystem health, as evidenced by three non-native grasses contributing to 50% of plant cover along the highly modified urban greenway. We suggest that actively fostering collaborations between floristic botanists and ecologists can create new insights into the maintenance of species diversity at the community scale.

## Introduction

What is the minimum sampling effort needed to adequately document plant community richness and composition? This question forms a fulcrum upon which multiple branches of plant science have revolved for decades [1–4]. We still do not have a universal solution, because the answer depends both on the characteristics of the sampled community and the goals of the researcher [5]. Further, there are several dimensions of biodiversity including richness, abundance, and evenness, each of which can manifest differently across temporal and spatial scales, as well as study systems [6,7]. Given the complexity, and increasing urgency, of describing Earth’s biodiversity, it is necessary to continue refining our sampling approaches.

Combining knowledge and research tools from multiple disciplines is one way to better describe complex systems [8–10]. One instance where collaboration remains elusive is between floristic botanists and plant ecologists. While these groups flank each other on the spectrum of biodiversity scientists, they are often siloed, in part because of their different disciplinary histories and research goals [11]. Here we explore the unique research lenses and sampling approaches that floristic botanists and plant ecologists use to describe plant community diversity. We then illustrate how these different approaches are complementary in describing diversity using research conducted along an urban greenway. We close by discussing the circumstances under which collaboration is likely to be most beneficial in this time of rapid biodiversity loss.

**Fig 1:**
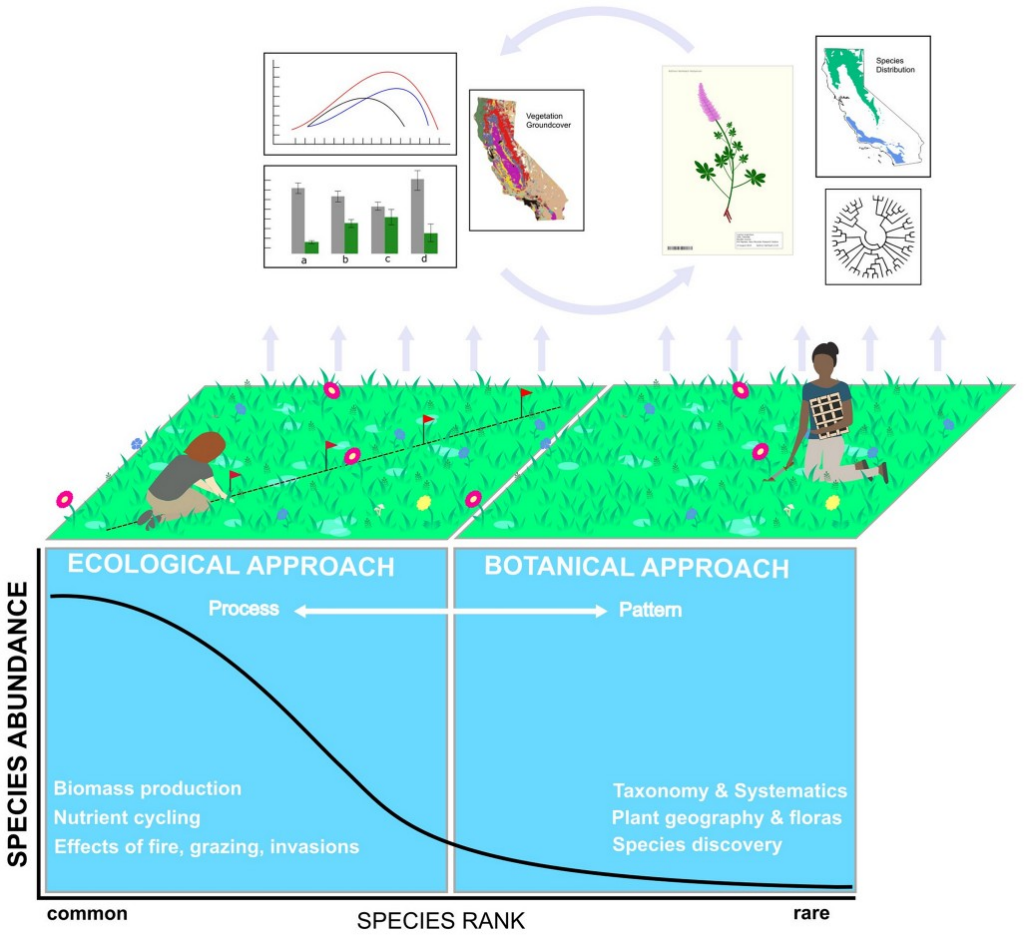
Conceptual diagram illustrating the different research foci, sampling approaches, and research outputs of floristic botanists and plant ecologists, placed within the context of the species-rank abundance curve. This curve typifies many plant communities in which a few species are common, while many species are rare. Plant ecology research often focuses on common species that drive ecological processes. Describing these processes requires transects or plots that allow for statistical hypothesis-testing to answer specific questions. This approach necessarily shrinks the area that can be sampled, causing species far out on the tail of rarity to be missed. Conversely, floristic botany focuses on species discovery, taxonomy, systematics, and the building of floras, which do not rely on statistically designed field sampling. This frees floristic botanists to search exhaustively for unique species in a study area, moving them farther out on the tail of rarity for a better estimate of a site’s “true” species pool. Each approach yields unique outputs. For example, the ecological approach may quantify contemporary relationships between plant relative abundances and their environment (top left of figure), while the floristic approach generates specimens, phylogenies, or floras with species distribution maps (top right of figure). The research outputs of each field could be more mutually informative (cycling arrows), e.g., if a collected specimen was linked to a plot-based estimate of that species relative abundance at the collection location.

Understanding plant community diversity requires documenting both pattern and process. We must first know which species exist, and where (i.e., taxonomic and biogeographic patterns), before we can determine why they exist there (i.e., ecological and evolutionary processes). Botanical specimens, which are routinely collected by floristic botanists, form the backbone of what we know about plant taxonomy and biogeography, from species discovery to the generation of exhaustive and meticulously vouchered local and regional floras [12] (Fig 1). While such data can in aggregate be used to test hypotheses about what shapes biodiversity, the primary data (i.e., physical specimens and species lists) are not designed to uncover site-level ecological processes. In contrast, ecological data are collected for the express purpose of answering a question or testing a hypothesis [13]. This often requires statistical design and analysis, including the use of replicated transects or plots, which are distributed in a manner that reduces biases in the resulting data [5,14,15]. Unavoidably, this requirement drastically shrinks the area that plant ecologists can sample [16], which has led to a multitude of papers concerned with determining “how much sampling is enough?” (with “enough” often approximated by the asymptote on a species accumulation or rarefaction curve) [17–20].

Floristic botanists, quite powerfully, are not tied to plots. They are free to make informed decisions about how much area must be covered to adequately sample a plant community of interest, which can often be completely traversed using an opportunistic sampling approach. Thus, botanical collectors can cover a considerably larger area than ecologists, thereby finding species further out on the tail of rarity (Fig 1) and generating a better estimate of the species pool in a given study area. Further, because botanists collect high-veracity specimens that are in flower or fruit, they usually have the structures needed for a species-level identification. Conversely, as part of their effort to reduce sampling biases, ecologists must identify everything captured in a plot regardless of phenological stage, potentially reducing the veracity of the identification or constraining it to the genus level (especially for graminoids). Still, ecologists typically capture the most abundant species in a community, with such species often driving ecological processes including nutrient cycling and response to disturbances like fire and grazing [21]. Importantly, only by using plot- or transect-based approaches can researchers move beyond documenting plant presence (richness) to quantitatively relating plant relative abundances to environmental variation.

Here we illustrate that the sampling approaches of botanists and ecologists are complementary, with the former providing critical context about the community species pool, and the latter layering on quantitative data for a subset of abundant species. We demonstrate the utility of implementing both approaches with a case study from an urban greenway in Colorado, USA. First, we compare species richness estimates generated using opportunistic sampling to those generated using ecological (line-point intercept) transects, assuming that the intensive floristic search will best approximate the “true” community species pool (sensu [28]). We then use rarefaction and species richness estimation to determine how closely the transect-based species-pool estimate reflects the larger species pool. This opportunistic versus bounded comparison of species pools is unusual (although not unique; see [5,22] for examples with mosses and lichens), with most vascular plant studies instead using plots of increasing size to estimate species pools [23]. Second, we explore whether the composition of the species pools captured by each approach leads to different ecological interpretations of the greenway’s flora. To this end, we compare the taxonomic coverage of each approach, as well as how species are distributed in relationship to functional group, biogeographic origin, floristic quality (based on Coefficients of Conservatism), and Wetland Indicator Status. Third, we integrate abundance estimates from the ecological transects into our interpretation of the greenway’s ecological condition to show that abundance is a desirable, if not necessary, complement to richness in any setting with applied conservation or management goals. Finally, we highlight areas of opportunity for collaboration between floristic botanists and plant ecologists.

## Methods

### Study system and field sampling

We sampled plant communities along the High Line Canal greenway, a 66-mile recreational trail that passes through 11 municipalities in the Denver-Metro area of Colorado. The trail runs alongside a 71-mile earthen canal (owned by Denver Water), which was excavated in the late 1800s to support agriculture and human settlement in what was historically native plains and foothills shrubland vegetation (see S1 Fig for a map of the greenway in relationship to EPA Ecoregions). The greenway thus represents a human-created waterway that is highly managed, yet supports a species pool that contains native flora (see [24] for habitat descriptions).

The greenway’s length (with the Canal’s inception located at 39.48362, -105.11293) is demarcated by mile markers that we used to generate a random subset of 45 locations at which the botanical and ecological field crews could synchronize their data collection. As is typical for collections-based floristic surveys, the botanical crew sampled exhaustively from early spring (7 May) through late summer (28 September) to capture early-, middle-, and late-season species. Starting at each of the 45 mile markers, the crew walked in the Canal’s downstream direction, searching the greenway for newly encountered species to collect and accession to the Kathryn Kalmbach Herbarium (KHD) at Denver Botanic Gardens [25]. Permission to collect plant specimens was provided by Denver Water. Most mile marker locations were sampled once during the inventory, but a few were revisited if they occurred in a vegetation type that would not be re-encountered later in the season at the other mile markers (e.g., mile markers zero and one at the inception of the Canal were the only locations in the foothills shrubland Ecoregion; S1 Fig).

We used a staggered sampling design in which 5 mile markers spanning the southwestern to northeastern extent of the Canal were sampled every other week from May to September. The floristic survey was carried out over 57 days, comprising 850 search-hours and an estimated distance covered of 42 miles (calculated from our daily starting and stopping waypoints logged with a GPS unit; S2 Fig). The botanical crew consisted of two botanists trained in the local flora and one to two additional non-botanists who assisted with specimen collection. All members of the crew searched for species within an ~50 to 75-foot-wide viewshed moving from the bed of the Canal, up the Canal bank, across the greenway trail, and over to the property line that marked the end of Denver Water’s ownership (S3 Fig). High-veracity (with identifying structures) herbarium specimens were accessioned for every species encountered during the floristic survey (numbering 1570 specimens, including duplicates; collections data available; 26). Identifications were made using [27–29].

The ecological sampling was carried out over 10 days, from May 22, 2018 to June 6, 2018, to capture a snapshot of plant communities around peak biomass. This method of deploying a concerted sampling effort over a short time period is common in ecological sampling, because it is often of interest to detect treatment differences that could be obscured by confounding time lags between sample dates (as opposed to the floristic botany goal of exhaustively delineating a species pool over time). At each of the 45 miles markers, we laid a 12 m × 2 m transect, the length of which captured habitat variation across the greenway corridor (S3 Fig). We used the line-point intercept method [30] to make field observations of plant species presence every 0.25 m along the 12 m transect (as well as bare ground, plant litter, and rocks, which we do not report herein) [31]. In the associated data set [32], the “first hit” was used to generate the reported percent cover estimates (number of hits per species per total number of hits), while the “second hit” was used to add species to our presence list. We also searched each of the two, one-meter-wide belt transects for additional species that were not encountered along the line-point transect. Voucher specimens were collected for the species encountered during the observational ecological sampling (collected outside the transects so as not to influence long-term sampling). However, given the short time period of the ecological sampling, not all specimens had flowers or fruits, and therefore were not of sufficient quality to be curated. All specimens were kept during the field season and subsequent analyses to facilitate identifications, but only higher quality specimens were accessioned to the herbarium [collections data available; 32]. Please note that one example specimen exists for potentially hundreds of field observations (i.e., each time a species was encountered along the transects).

We chose the line-point method as the most appropriate for our system, with its narrow and steep canal bank that could not accommodate other plot designs. Additionally, our aim with the ecological transects was to estimate not only species presence, but also composition. For questions about composition, the line-point method is highly repeatable across individuals and rapidly deployed, thereby maximizing sampling replicates across many locations in a single season [30]. Such transects will capture fewer species than other methods (e.g., Modified-Whitaker plots); however, any bounded sampling approach will cover considerably less area than can be achieved with opportunistic sampling based in the floristic tradition of using the habitat itself as the sampling unit [5]. Importantly, we note that it was not our goal to equalize the temporal or spatial scales of the two sampling approaches (which in our experience is not often done in practice), but rather to sample in a manner that is broadly consistent with collections-based versus ecological disciplines. The particulars of our comparison, such as the sampling window and the use of transects rather than any number of plot types, contextualize the results.

### Community species pools and ecological metrics

We first estimated the species pools captured by the collections-based floristic and quantitative ecological sampling approaches, and then compared the pools in ecologically meaningful ways. Species pools are hierarchical and scale dependent, having been variously defined, but they are typically partitioned from larger scale (regional pools), to mid-scale (local pools), to smaller scale (actual or community pools). Here, we define a community species pool according to [33] as “the set of species present in a target community,” with our target community being the urban greenway. We consider the species found during the intensive floristic sampling as a best estimate of the greenway’s “true” community species pool and expect that the ecological transects will capture a subset of this larger pool. (We acknowledge that even the intensive floristic sampling will not capture the true pool, but the goal is to employ realistic sampling schemes used in botanical floristics and plant ecology to see how they compare).

After delineating the species pools, we chose several ecologically informative metrics to compare them: species distributions among families; plant functional group based on longevity and growth form; floristic quality (based on Coefficients of Conservatism or *C* values) [34]; Wetland Indicator Status [35]; and biogeographic origin (assigned as native or introduced to Colorado according to [27], and if introduced, whether it is cultivated). Plant functional groups are extensively used to aggregate large numbers of species into a few classes that are expected to respond similarly to changes in their environment, or to similarly affect their environment [36–38]. Floristic Quality Analysis is often used in the conservation realm to assess an area’s conservation value [34]. Sites with high floristic quality are relatively pristine, having departed little from the disturbance regime that existed prior to European settlement. Related to this, species in a community can be ranked on a scale of zero to 10 according to their “conservatism,” or their fidelity to habitats that are more (or less) degraded by human use. Species that can only persist in undegraded, native habitats are assigned high *C* values, while ruderal species, which can withstand substantial degradation, are assigned low scores (rankings below adapted from [39]).

0–3: Introduced species (always = 0), plus native species that occur in moderately to highly degraded sites (1–3)
4–6: Native species that show some affinity to natural areas and are often dominant or are present across a wide range of habitats and environments
7–8: Native species associated mostly with natural areas, but that can sometimes persist in degraded habitat
9–10: Native species that tolerate very little or no habitat degradation

Wetland Indicator Status ranks species according to their dependence on saturated soils, or wetland conditions. This metric is meaningful in our study system because the Canal represents a novel waterway in an otherwise semi-arid landscape. The status rankings are as follows. 1) Obligate Wetland: almost always a hydrophyte, rarely found in uplands; 2) Facultative Wetland: usually a hydrophyte but occasionally found in uplands; 3) Facultative: commonly occurs as either a hydrophyte or non-hydrophyte; 4) Facultative Upland: occasionally a hydrophyte, but usually occurs in uplands; 5) Upland: rarely a hydrophyte, almost always found in uplands [40]. Our study area occurs on the interface of the Western Mountain Valleys and Coasts and Great Plains regions, which can occasionally have different wetland indicators assigned for the same species. If there was a discrepancy between regions, we chose the more hydrophilic option to generate a conservative list in terms of species reliance upon water.

### Data analysis

We calculated the expected number of species in our pooled samples using species rarefaction. The rarefaction curve was produced from 1000 random resamples drawn without replacement from the pool of species in the transects. We extrapolated out to 1.5X the original sample size (68 transects), as extrapolation past doubling or tripling of the reference sample size is not recommended due to increased uncertainty [41]. Asymptotic species richness was estimated using the Chao2 estimator in EstimateS version 9.1.0 [42] using the incidence of each species within each sampling transect.

We used Pearson’s Chi-square tests of independence ([43]; chisq.test in R v. 3.6.2) to explore whether the floristic and ecological sampling approaches generated different distributions of species in relationship to taxonomic coverage, functional groups, *C* values, Wetland Indicator Status, and biogeographic origin. For each data set (floristic and ecological) we summed species frequencies from the 45 mile markers to alleviate low cell counts within mile markers. When the sampling approaches generated significantly different (*P* ≤ 0.05) distributions of species among groups, we used the adjusted standardized residuals to assess which groups contributed to the disparity (with residuals exceeding an absolute value of approximately 2 considered important; [44]). We could not assign functional groups to nine species that were only identified to the level of genus. We also could not assign *C* values to a subset of species that did not have them available (n = 18 species or 4% of all collections and six species or 5% from transects). The same was true for Wetland Indicator Status, in particular for cultivated species, which are not assigned this type of indictor (n = 76 species, or 17% of all collections, and 10 species, or 8% from transects).

For the purpose of assessing species composition along the greenway, we calculated abundance using percent cover from the ecological transects. Abundance estimates were calculated by taking the number of hits per species divided by the total number of hits sampled over the entire Canal [raw data available; 31].

## Results

**Fig 2:**
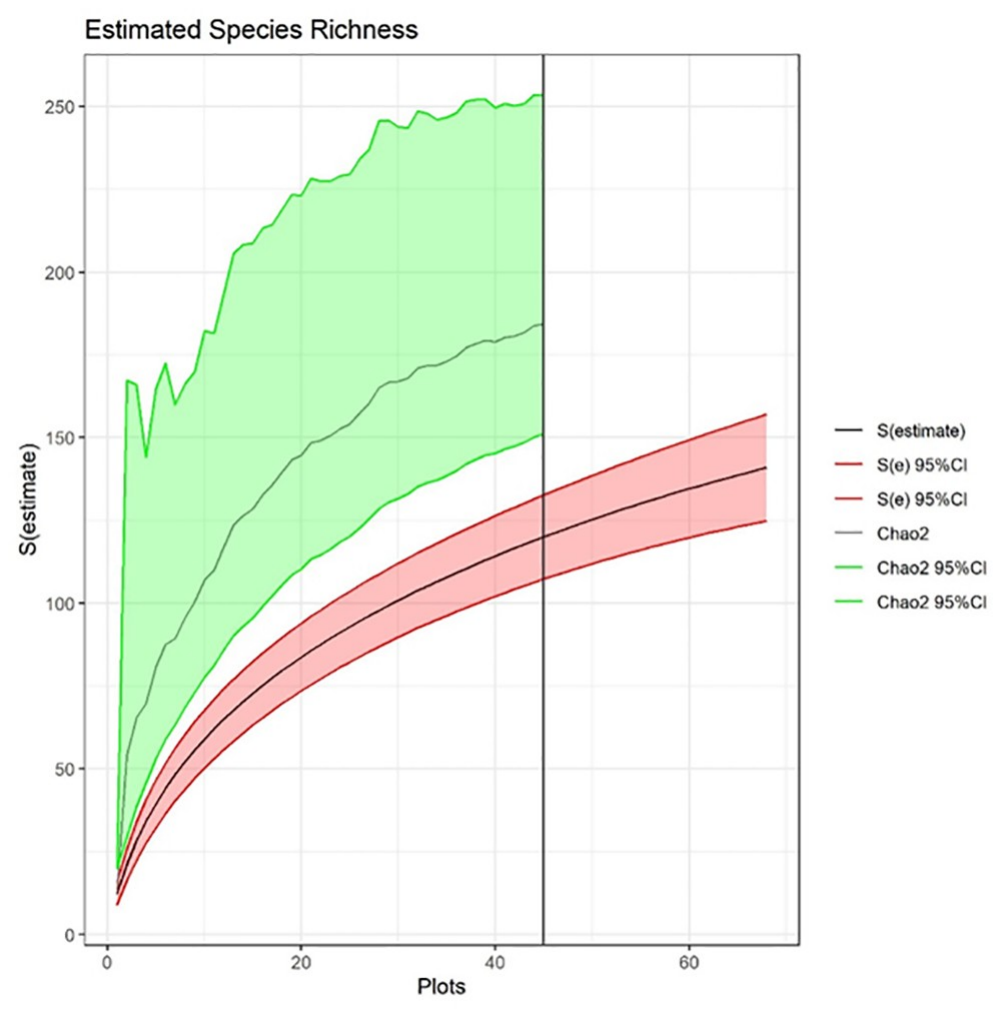
Species richness estimated using transect-based rarefaction (black line shown with 95% CI in red shading) based on 45 sampled transects and extrapolated up to 68 transects (values after the vertical black line). An asymptotic richness estimate was calculated using the Chao2 estimator (grey line with 95% CI in green shading).

We found 452 species using the opportunistic sampling approach used in floristic botany [collections data available; 45] and 126 species using ecological transects (see S1 Table for full species list). Species richness modeled from the transect data underestimated the floristic estimate by 41% (mean = 184; 95% CI lower bound = 151; 95% CI upper bound = 253; Fig 2). This marked underestimate manifested despite using the Chao2 estimator, which statistically accounts for the fact that uncommon species will likely be missed. The species rarefaction curve showed that a 51% increase in our sampling effort, from 45 to 68 transects, would only capture 31% of the species pool observed during the opportunistic sampling (mean = 141; 95% CI lower bound = 125; 95% CI upper bound = 157; Fig 2). While neither the species rarefaction curve nor the Chao2 estimator reached a definitive asymptote (although they were distinctly leveling off), our transect sampling effort was based on what we could reasonably achieve with the available financial and personnel resources. We expect that other researchers are similarly constrained in most applied situations.

**Fig 3:**
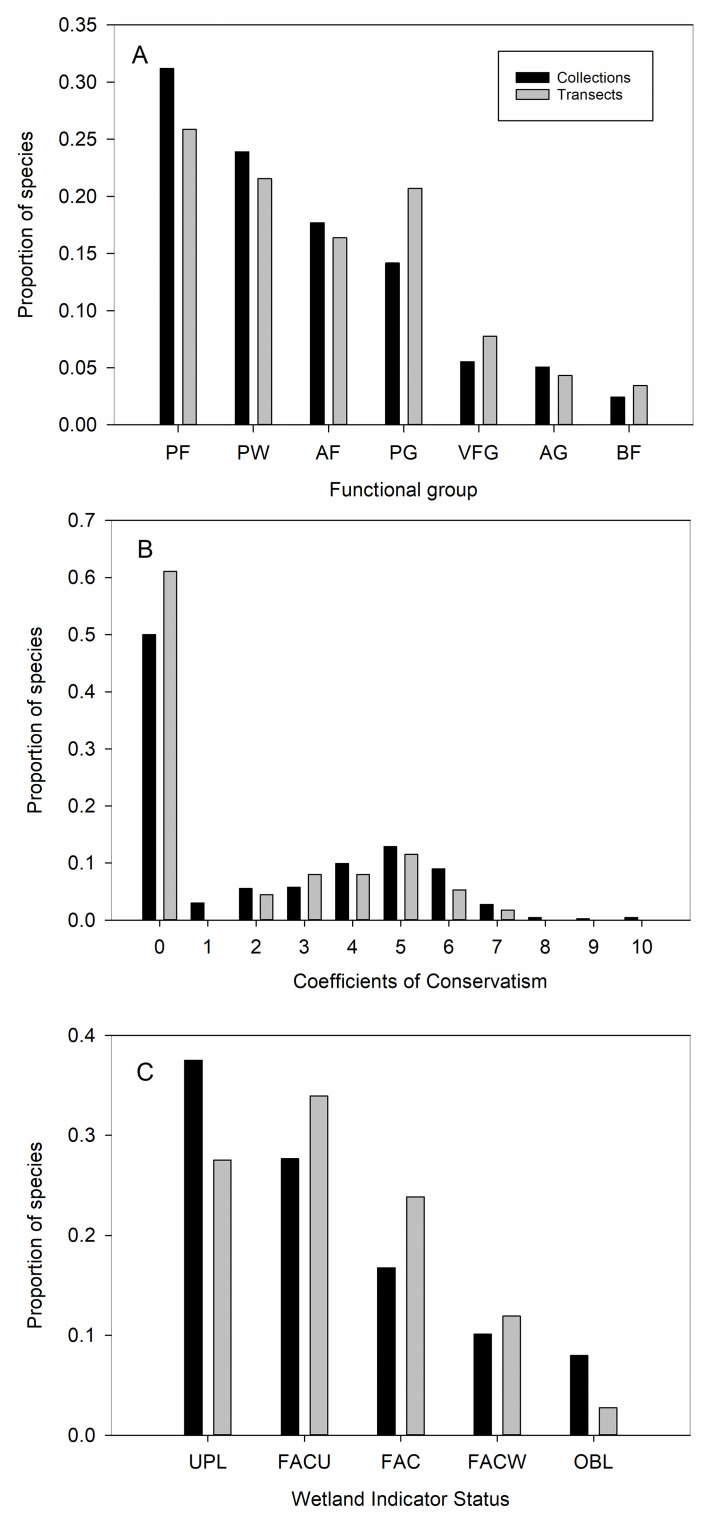
**A-C.** Distribution of species captured by the floristic botany (collections) and ecological (transects) sampling approaches in relationship to functional groups, floristic quality (based on Coefficients of Conservatism or *C* values), and Wetland Indicator Status. The distribution of species among functional groups and *C* values did not significantly differ, while the distribution among wetland indicator status differed. Functional groups: PF = perennial forb; W = woody (and perennial); AF = annual forb; PG = perennial grass; VFG = variable forbs and grasses (annual to short-lived perennials; includes only two species of grasses); AG = annual grass; BF = biennial forb. Wetland Indicator Status: OBL = obligate wetland; FACW = facultative wetland; FAC = facultative; FACU = facultative upland; UPL = upland. See text for statistics and definitions of *C* values and wetland indicators.

The taxonomic coverage of the ecological versus botanical sampling became less complete moving hierarchically from family, to genus, to species (with transects capturing 50% of families [39 versus 78], 36% of genera [103 versus 289], and 28% of species [126 versus 452]; see S4 Fig for species distributions among families). The distribution of species among functional groups was statistically similar for the two sampling approaches (Fig 3A; χ^2^ = 5.6, df = 7, *P* = 0.59), although perennial grasses were weakly over-represented (by 6%) along transects, reflecting the pattern found for family distributions (see Poaceae, S4 Fig). The floristic quality of the greenway, based on the distribution of species among *C* values (Fig 3B; χ^2^ = 9.9, df = 10, *P* = 0.45), did not differ significantly between the two sampling approaches. Wetland Indicator Status significantly differed (Fig 3C; χ^2^ = 10.6, df = 4, *P* = 0.03), with transects underestimating obligate wetland species by 5% (adjusted residuals = -2.05) and upland species by 10% (adjusted residuals = 2.0) relative to the larger species pool. In terms of biogeographic origin, the transects over-estimated (although non-significantly) the proportion of introduced species (55%) relative to the opportunistic sampling (46%; χ^2^ = 2.75, df = 1, *P* = 0.10). The composition of introduced species captured by the two approaches differed in an important respect, with the floristic approach capturing a substantial number of uncommon, non-native garden cultivars (79 cultivars out of 208 introduced species, or 38%) that the transects missed (four cultivars out of 68 introduced species, or 6%).

**Fig 4:**
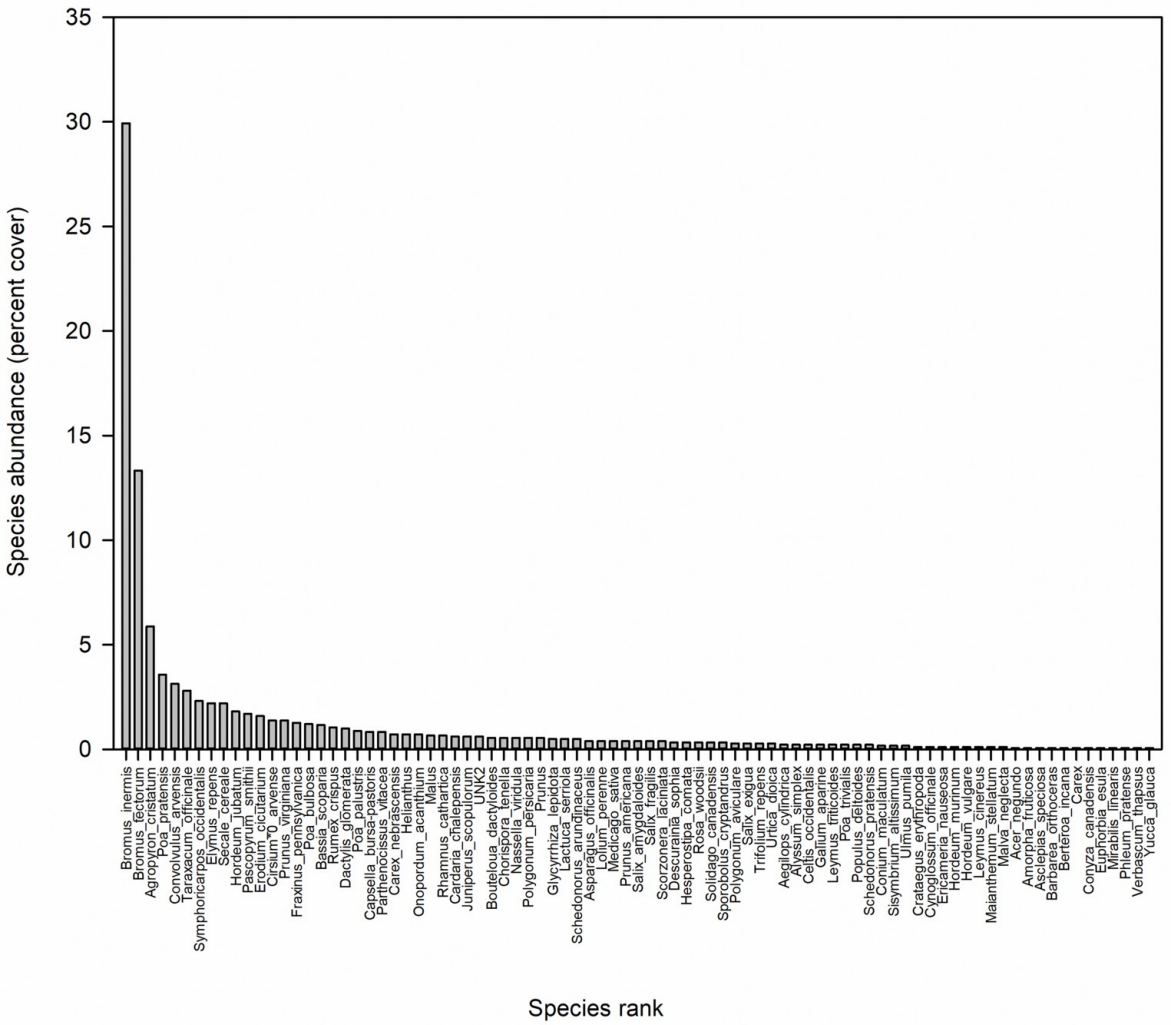
Species relative abundances (percent cover) estimated using the ecological transects showing that only three species (the introduced grasses *Bromus inermis* L., *Bromus tectorum* L., and *Agropyron cristatum* (L.) Gaertn.) made up nearly half of the greenway’s percent cover during the 2018 sampling window. Compare with the conceptual diagram of the species-rank abundance curve in Fig 1.

In terms of abundance, the greenway revealed a typical species-rank abundance curve in which a few species dominated, while a long tail of uncommon species contributed to species richness (Fig 4). The three most abundant species, which comprised ~50% of plant cover during our sampling window, were three non-native grasses: *Bromus inermis* L. (smooth brome), *Bromus tectorum* L. (cheatgrass), and *Agropyron cristatum* (L.) Gaertn. (crested wheatgrass).

## Discussion

Here we illustrate that sampling approaches from floristic botany and plant ecology capture complementary dimensions of biodiversity. As hypothesized, opportunistic sampling generated a markedly more robust (nearly four times larger) empirical estimate of the greenway’s community species pool than did the transects. Yet, the transects revealed that only three species of introduced grasses comprised 50% of plant cover at the time of sampling. Generating this critical indicator of the greenway’s ecological condition necessarily traded off with a fuller assessment of the species pool. This long-acknowledged trade-off is typically addressed by using rarefaction and accumulation curves and species richness estimators based on transect or plot data [46]. Our findings suggest that for sampling designs with limited areal coverage, such tools may not adequately capture the long tail of uncommon species that contribute disproportionately to species richness [13,47,48]. This issue was raised by Heilmann-Clausen and Læssøe [49], who clarified that species accumulation curves and species richness estimators address “how many species will be recorded if [a particular] sampling regime is followed in perpetuity or extended to cover all available habitat,” rather than telling the size of the species pool in the system. Related, Newmaster et al. (2005) illustrated that rarefaction and Chao estimates of common forest moss species reached an asymptote at small sample sizes (25 plots), while estimates of richness for rare species never leveled off. Thus, the very transect- or plot-based studies that often rely on estimator tools to determine the completeness of sampling likely produce underestimates of the true species pool [50,51]. Of course, many ecological questions can be rigorously answered without exhaustive documentation the species pool. Still, it is worthwhile to address these interpretive nuances when presenting results based on species estimators.

The question then becomes whether and when the transect-based sampling constraint, and any attendant underestimate of the species pool, affects how the ecology of the sampled area is interpreted (at least in terms of species richness). To assess this, we grouped species into ecologically meaningful categories to reduce complexity and uncover general patterns that are independent of species identity per se [52,53]. We found that based on functional groups and floristic quality (i.e., *C*-values), the ecological condition of the greenway appears similar between the two species pools. This comparable delineation of functional groups is especially desirable given that functional group identity and diversity are routinely used to gauge community response to disturbances such as fire and biological invasions [54,55]. In our system, functional traits such as longevity and woodiness (and attendant traits like rooting depth) likely shape soil and hydrologic conditions along the Canal banks. In terms of floristic quality, both sampling approaches captured the bell-shaped distribution of native species and similarly indicate that few conservative (*C*-value of 7 or more) species remain along this highly modified urban corridor. These findings corroborate previous work showing that floristic quality performs well when using transects or plots, because mean *C*-values are less dependent on the area sampled than is species richness [34].

While transects robustly captured patterns associated with functional groups and floristic quality, differences between the sampling approaches arose for Wetland Indicator Status and the presence of uncommon cultivated species. In particular, patchily distributed wetland areas and the obligate wetland species they harbor went largely undetected by the transects. This is a non-trivial miss in a Canal system where episodic drought and various management strategies strongly affect the hydrologic regime and thus persistence of sensitive wetland areas. Transects also failed to detect the nascent incursion of garden plants from adjacent private properties onto the Canal banks, potentially hindering Early Detection and Rapid Response (EDRR) management interventions [56,57]. These examples illustrate that without knowing the more complete species pool, the ability to thoroughly uncover sensitive ecological conditions along the greenway would be hampered. Still, if only species richness from the floristic inventory were used to assess the greenway, the high abundance of non-native grasses would go unreported. Without these abundance data, it would be impossible to assess costs associated with potential control or revegetation efforts, as well as to relate these dominant grasses to ecological processes of interest. For example, we are currently asking how the implementation of green stormwater infrastructure will affect vegetation along the Canal banks, and in turn, how in situ vegetation will affect stormwater infiltration, retention, and removal of pollutants. We are now positioned to take a two-pronged approach to this question by exploring how the most abundant species might shape stormwater dynamics, while also integrating information about the identity and location of uncommon species likely to be particularly responsive to hydrologic changes (e.g., obligate wetland species) and disturbance from infrastructure installations (e.g., establishing individuals of ruderal non-natives).

Broadening out from our greenway example, when should floristic botanists and ecologists develop on-the-ground collaborations to better describe contemporary biodiversity? We suggest that any question about the maintenance of community-level species diversity would benefit from a paired approach, as it is ultimately the interplay of local (competition, predation, microenvironmental variation) and regional (immigration and extinction) processes that shape biodiversity [58–60]. To better integrate across spatial scales, it would be powerful to link quantitative data from bounded sampling to floristically based best estimates of the community species pool that functions as the backdrop for species immigration into embedded transects or plots. (While many terrestrial plant studies sample hierarchically across plot sizes to infer species pool sizes, they fall short of breaking free of plots to approach a comprehensive site- or habitat-level survey; [23]). Moreover, pairing floristic and ecological approaches addresses the call for increased metric complementarity in assessing biodiversity [61]. In particular, the historically heavy reliance upon species richness as a sole indicator of ecosystem health or biodiversity change has proven insufficient, as it fails to capture changes in other key phenomena such as species turnover and changes in species relative abundances [61,62]. Re-imagining floristic surveys of species richness as integral components of hypothesis-driven ecological work that uses other metrics can lead to new insights.

For example, to achieve metric complementarity in a restoration context, a floristic inventory could be used to assess the feasibility of passive restoration (which depends on the community species pool; [63,64]), while paired ecological sampling could quantify the degree of habitat degradation and monitor effectiveness of restoration efforts. Invasive species management would also benefit from a combined sampling approach, where initial arrivals of rare introduced species to an area are captured during unbounded floristic sampling bouts, while the spread and population biology of already established populations are monitored using transects or plots. Indeed, integrating researchers versed in alpha taxonomy is critical in invasion biology, as the resolution of taxonomically challenging groups, including those that hybridize, is critical to proper ecological interpretation [10]. A further boon of integrating floristic botany into settings where the species pool is of interest is excellent temporal sampling of early- to late-season bloomers, which can be missed using the “peak biomass” approach typical of ecological sampling [65]. Moreover, validation of transect- or plot-based data with vouchered specimens is tantamount to institutional knowledge that can be readily accessed by all researchers who carve out projects from a particular locale. The long-standing specimen and data curation practices used in natural history collections have achieved a level of standardization and data-sharing not yet realized in the ecological realm. However, improvements are being made in this arena, such as the application of the event-based Darwin Core data standard when publishing ecological data, as we have done herein [26,45].

To most informatively link plot- and site-scale diversity data, best practices would be to pair the replicable plot data with equally replicable floristic sampling of the community species pool [e.g., 22,62,63]. Collections-based floristic botany has not fully adopted standardized field sampling practices across individual collectors [66], which can limit the use of collections data for analyzing hypothesis-driven questions about community-level change in diversity [67]. The value of specimens and species lists generated using the opportunistic sampling approach could be increased by reporting the spatial extent of the surveyed area [68], as well as the intended goal of the collection event (e.g., an exhaustive inventory [implying species absence] versus targeted sampling based on an investigator’s taxa of interest). Such reporting practices can be achieved within the Ecological Metadata Language standard contained within a data package [69] and would provide additional context for downstream uses of aggregated data (e.g., species pool estimates derived from geo-referenced specimen databases) [70]. However, it must be considered that standardizing collections data (or at least reporting accurate areal coverage of survey sites) requires some degree of bounding that is both time-consuming and at odds with maximizing the number of species encountered [68]. Thus, it may only be worth bounding collections-based sampling when the specific question calls for it (e.g., quantifying immigration into experimental plots from the surrounding species pool).

Our experience is that physical collections are not often considered by ecological principal investigators as essential to their field protocols (despite substantial movement in this direction by, e.g., the National Ecological Observatory Network). This is partly because it is no small task to integrate the disparate training, project planning, data curation, and analyses implemented in floristic botany and ecology [71–73]. Thus, cross-disciplinary partnerships across non-profit, governmental, and academic institutions are key. We suggest that ecologists reach out to their campus or regional herbaria, connect with curators and collections managers, and dedicate a line item in their research budgets for vouchered floristic (or faunistic) surveys of their study sites (see [74] for discussion of under-funding in collections-based research). We similarly suggest that curators and collections managers build relationships with ecology principal investigators and members of their labs, sharing their skills as integral assets to be included a priori into the proper design of biodiversity-focused ecological fieldwork (i.e., it is imperative to quash the “end-user mode” attitude that views botanists as simply providing identification services to those who use their keys and field guides [75,76]). For example, gaps in biodiversity data, including both species discovery and ecological monitoring, are high in tropical relative to temperate ecosystems [77,78], and would thus be best addressed by teams of floristic botanists and ecologists. As large-scale digitization of collections data has revealed, there are myriad, previously unimaged ways that natural history collections can inform ecological questions [79]. We believe our understanding of biodiversity 100 years from now can only benefit from thoughtful co-exploration of today’s ecosystems by floristic botanists and ecologists.

## Supporting information

S1 Fig Map of High Line Canal greenway in relationship to US EPA level IV Ecoregions. The “Character Zones” overlaid on the Ecoregions represent large-scale variation from the southwest to the northeast of the greenway, characterized by a transition from foothills to plains habitat, which is in turn overlaid by different degrees of land use intensity. These habitat and land use factors shape the “character” of the greenway, as the viewshed changes in relationship to topography, type and density of vegetation, and the type and density of surrounding development.
(TIF)

S2 Fig Map of greenway extent covered during the opportunistic floristic sampling as measured by daily starting and stopping waypoints logged with a GPS unit. The “Character Zones” overlaid on the satellite imagery represent large-scale variation from the southwest to the northeast of the greenway, characterized by a transition from foothills to plains habitat, which is in turn overlaid by different degrees of land use intensity. These habitat and land use factors shape the “character” of the greenway, as the viewshed changes in relationship to topography, type and density of vegetation, and the type and density of surrounding development.
(TIF)

S3 Fig Schematic of ecological transect orientation in relationship to High Line Canal greenway. (TIF)

S4 Fig Species distributions among the most common families observed along the High Line Canal greenway in Colorado, USA, using floristic botany (opportunistic) and ecological (transect-based) sampling approaches. (TIF)

S1 Table List of species found using the floristic collections-based versus ecological transect-based sampling approaches. (XLSX)
