# Supplementary figures and images for: Combining botanical collections and ecological data to better describe plant community diversity
Source: PLoS One. 2021 Jan 7;16(1):e0244982. doi: 10.1371/journal.pone.0244982 (PMC7790410; doi:10.1371/journal.pone.0244982)

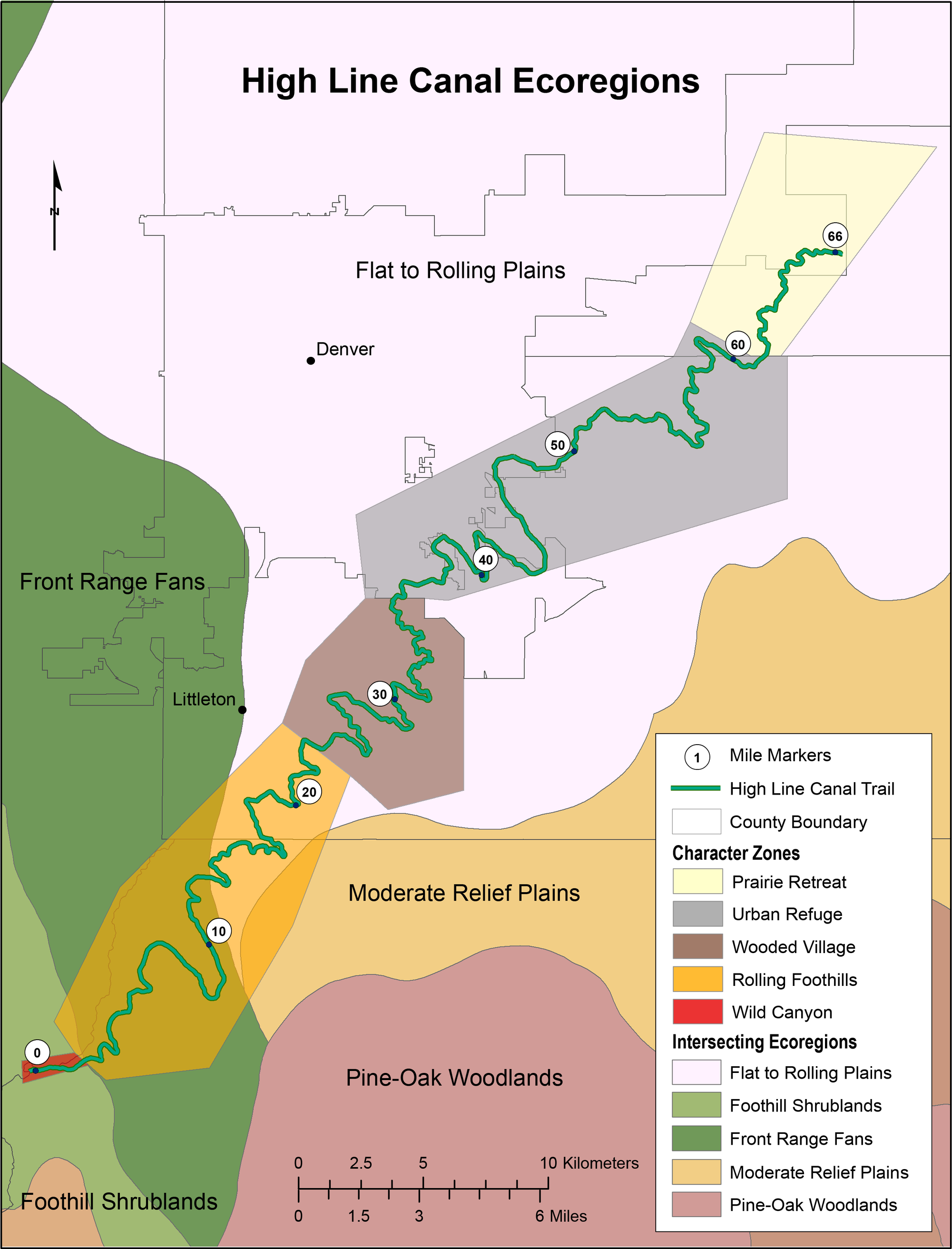

Supplement: S1 Fig — The “Character Zones” overlaid on the Ecoregions represent large-scale variation from the southwest to the northeast of the greenway, characterized by a transition from foothills to plains habitat, which is in turn overlaid by different degrees of land use intensity. These habitat and land use factors shape the “character” of the greenway, as the viewshed changes in relationship to topography, type and density of vegetation, and the type and density of surrounding development. (TIF) [file pone.0244982.s001.tif]

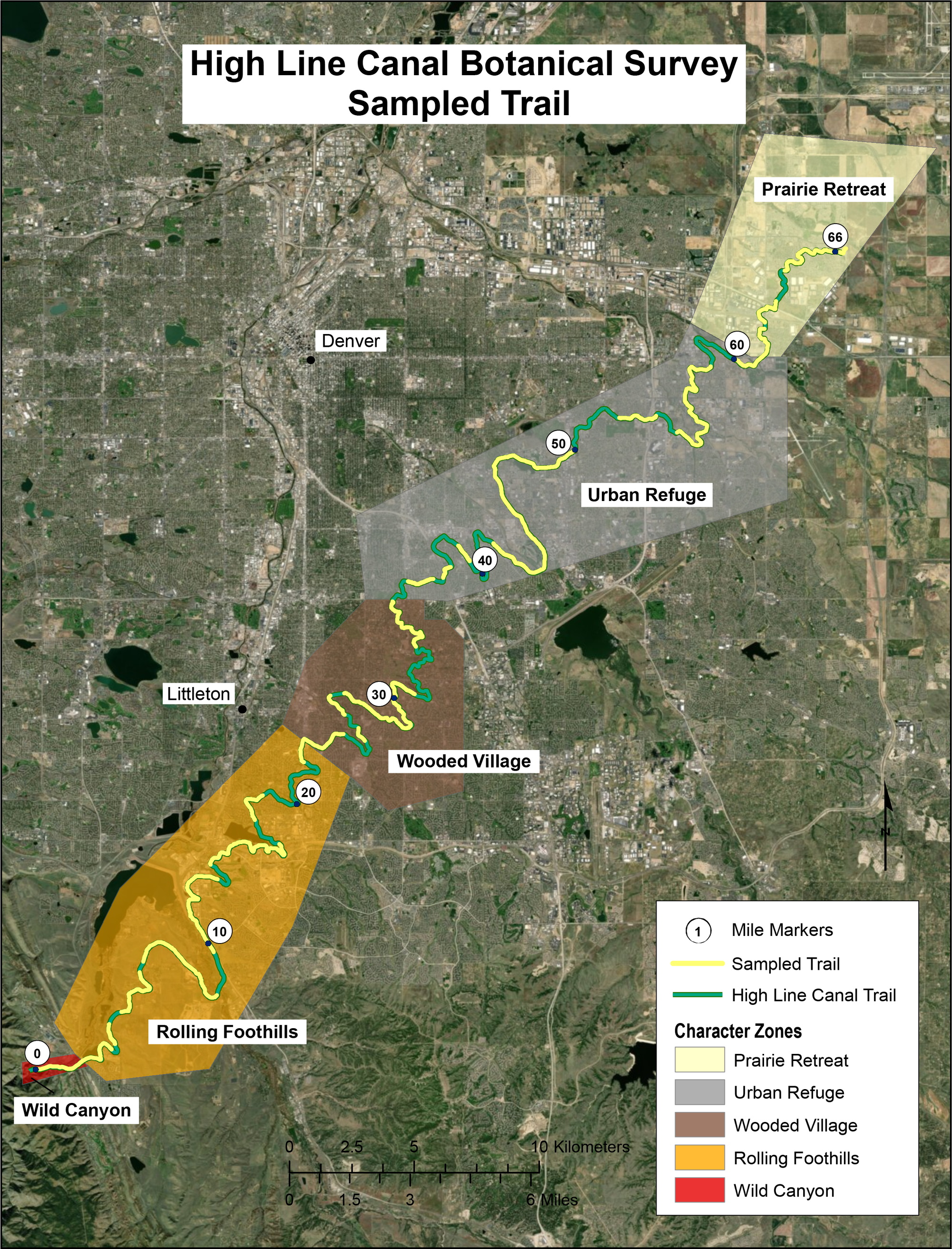

Supplement: S2 Fig — The “Character Zones” overlaid on the satellite imagery represent large-scale variation from the southwest to the northeast of the greenway, characterized by a transition from foothills to plains habitat, which is in turn overlaid by different degrees of land use intensity. These habitat and land use factors shape the “character” of the greenway, as the viewshed changes in relationship to topography, type and density of vegetation, and the type and density of surrounding development. (TIF) [file pone.0244982.s002.tif]

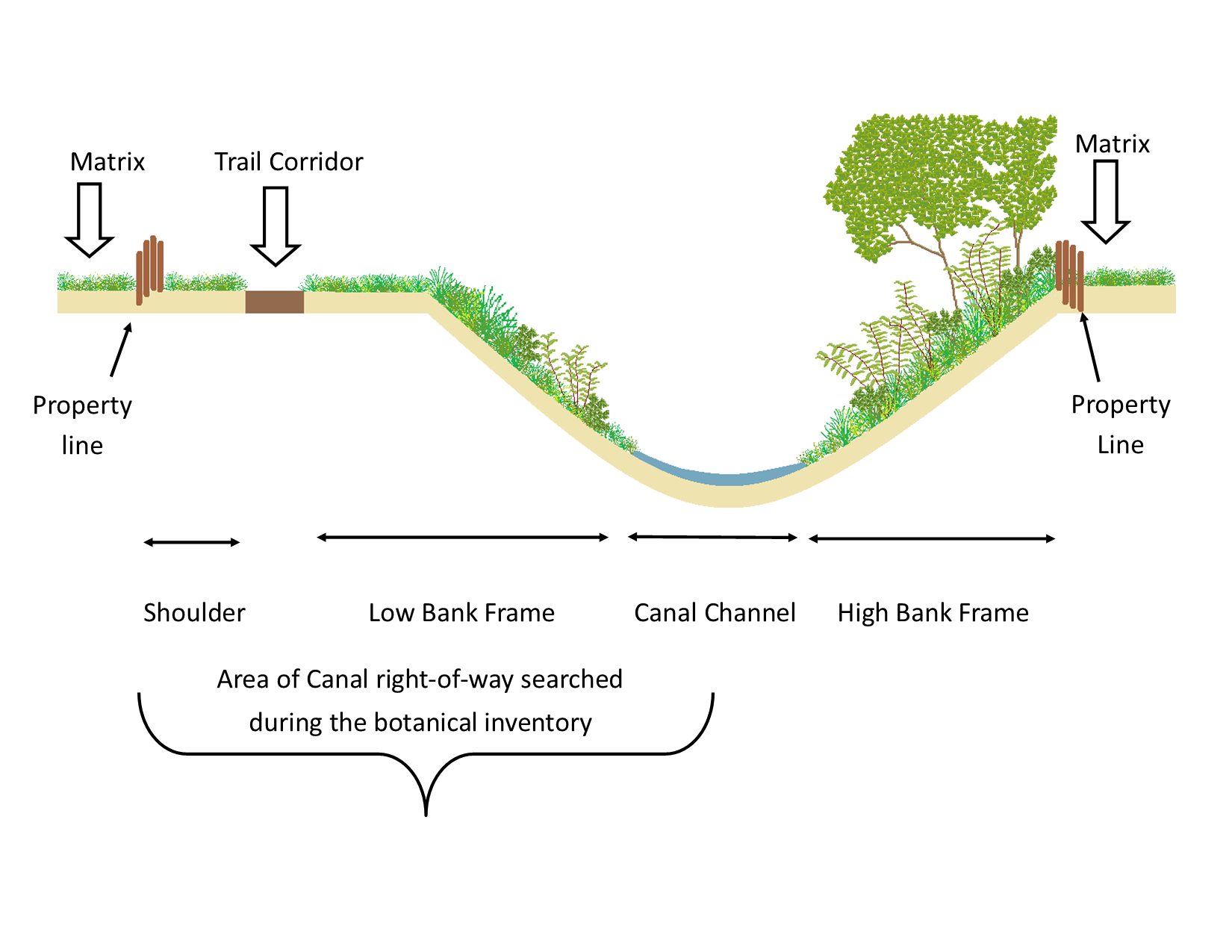

Supplement: S3 Fig — (TIF) [file pone.0244982.s003.tif]

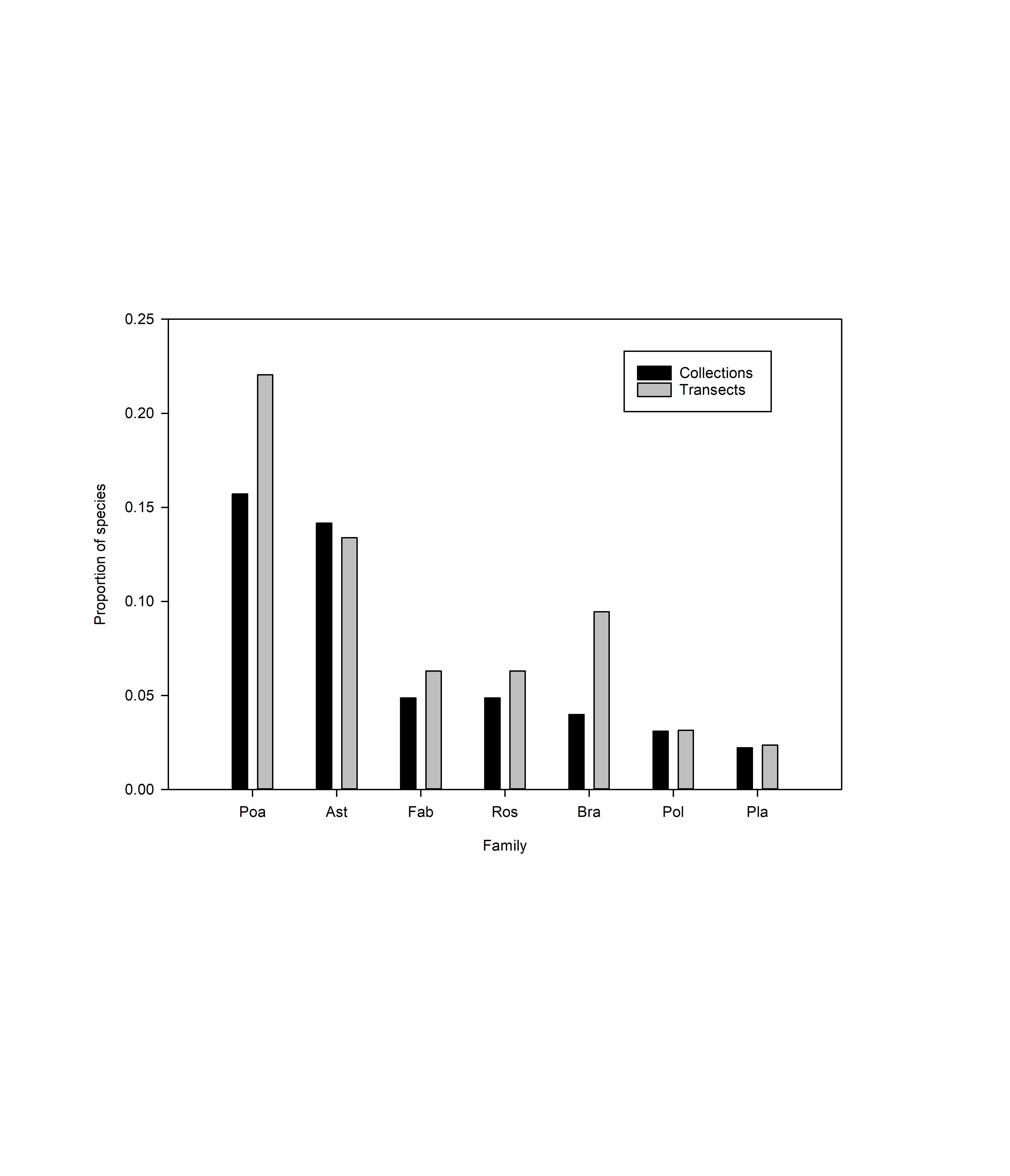

Supplement: S4 Fig — (TIF) [file pone.0244982.s004.tif]
